# African COVID-19 special issue introduction

**DOI:** 10.1017/S0950268821002739

**Published:** 2021-12-27

**Authors:** Norman Noah, Michael Edelstein

**Affiliations:** London School of Hygiene & Tropical Medicine, London, UK

Africa has been a continent vulnerable to outbreaks of both old and new viruses in recent decades, with ‘more than 100 significant public health emergencies annually’. When COVID-19 arrived in February 2020, having been imported mainly from Europe, it was perhaps even more unwelcome there than anywhere else in the world. As in other continents, it spread rapidly throughout Africa.

The WHO Health Emergencies Programme (WHE) had been formed to detect and respond to public health emergencies. *Epidemiology and Infection* commissioned the Emergencies Preparedness and Response Programme in the Regional Office for Africa to document the successes and challenges encountered in the first year of the fight against the COVID-19 pandemic. The collection illustrates the outstanding efforts from the WHO regional office, country offices and member states to deal with COVID-19. This supplement, produced in record time, is the result. The *Epidemiology and Infection* Editorial board is particularly honoured to have the series introduced by an editorial from Dr Matshidiso Moeti, WHO director for the Regional Office for Africa.

*Epidemiology and Infection* is most grateful to Dr Benido Impouma and his colleagues for working so hard to produce this fine collection of papers.

Norman Noah

*Editor-in-Chief*

